# Patterns, levels and correlates of self-reported physical activity in urban black Soweto women

**DOI:** 10.1186/1471-2458-14-934

**Published:** 2014-09-08

**Authors:** Philippe Jean-Luc Gradidge, Nigel J Crowther, Esnat D Chirwa, Shane A Norris, Lisa K Micklesfield

**Affiliations:** Centre for Exercise Science and Sports Medicine (CESSM), Faculty of Health Sciences, University of the Witwatersrand, Johannesburg, South Africa; MRC/Wits Developmental Pathways for Health Research Unit, Faculty of Health Sciences, University of the Witwatersrand, Johannesburg, South Africa; Department of Chemical Pathology, National Health Laboratory Service, Faculty of Health Sciences, University of the Witwatersrand, Johannesburg, South Africa

**Keywords:** Physical activity, Sitting time, Women, Black African, Cardiometabolic disease, South Africa

## Abstract

**Background:**

Urban black South African women have a high prevalence of non-communicable diseases such as obesity and type 2 diabetes. The aim of this study was to assess the physical activity patterns of a cohort of middle-aged urban-dwelling black African women and to determine if physical activity is associated with anthropometric measures and metabolic outcomes in this population.

**Methods:**

Physical activity and sitting time were assessed using the Global Physical Activity Questionnaire (GPAQ) in a cross-sectional study of 977 black African women (mean age 41.0 ± 7.84 years) from the Birth to Twenty study based in Soweto, Johannesburg. Anthropometric outcomes were measured and fasting blood glucose, insulin and lipid profile were analysed to determine metabolic disease risk and prevalence.

**Results:**

Sixty-seven percent of the population were classified as active according to GPAQ criteria, and the domain that contributed most to overall weekly physical activity was walking for travel. Only 45.0% of women participated in leisure time activity. The prevalence of metabolic syndrome in this sample was 40.0%, and the prevalence of overweight and obesity was 29.2% and 48.0%, respectively. Women who reported owning a motor vehicle walked for travel less, and participated in more leisure-time activity (both p < 0.01), while women who owned a television reported significantly lower moderate-vigorous physical activity (MVPA), and walking for travel (both p < 0.01). Sitting time (mins/wk) was not different between the activity groups, but was associated with triglycerides and diastolic blood pressure. Total physical activity was inversely associated with fasting insulin, and physical activity in the work domain was associated with fat free soft tissue mass.

**Conclusions:**

The findings of this study show that the majority of urban dwelling black South African women are classified as physically active despite a high prevalence of obesity and metabolic disease risk factors. Sitting time had detrimental effects on both triglyceride levels and diastolic blood pressure whilst total physical activity attenuated fasting insulin levels. As walking for travel is a major contributor to physical activity, future research should attempt to determine whether the intensity of this activity plays a role in the prevention of cardiometabolic diseases.

**Electronic supplementary material:**

The online version of this article (doi:10.1186/1471-2458-14-934) contains supplementary material, which is available to authorized users.

## Background

South Africa is a middle income country experiencing epidemiological transition due to rapid urbanisation. Nearly two thirds of the population live in urban areas, with the urban poor carrying the greatest risk of mortality from modifiable non-communicable diseases (NCD) [1–3]. Physical inactivity is accepted as one of the key chronic disease risk factors with 2008 estimations showing that physical inactivity was responsible for approximately 9% of deaths globally [4], however data on its prevalence within developing countries are sparse [5].

South Africa is estimated to be the third most physically inactive country in Africa, with more than half of the population being physically inactive (51.1%) [4]. Studies have shown that South African women are more inactive than men [5, 6], suggesting that they may be at a higher risk for chronic diseases resulting from physical inactivity [7]. A study from the South African Comparative Risk Assessment Collaborating Group has shown that in adult South African women, an estimated 27.7% of colon cancer, 22.7% of ischaemic strokes, 20.1% of type 2 diabetes mellitus, 30.5% of ischaemic heart disease, and 16.5% of female breast cancer are attributed to inactivity [8]. Black women in South Africa have the highest prevalence of obesity [9], which may partly be influenced by socioeconomic status [10], but may also be related to physical activity since it has been observed that black females in South Africa have significantly less total energy expenditure than white women [11]. Urban dwelling black South African females have recently been confirmed to be less physically active than rural women who usually accumulate higher levels of physical activity by participating in subsistence related activity and walking [12]. Data from the Transition and Health during Urbanisation of South Africans (THUSA) study has shown that physical inactivity is associated with obesity outcomes in black South African women [13]. Sitting time, a proxy measurement for sedentary behaviour, increases the risk for all-cause mortality independent of physical activity time [14]. Only a few studies have reported the estimated prevalence of sedentary behaviour in black South African females [12, 15]. These studies showed that rural women were significantly more sedentary than rural men [15], and that urban women were significantly more sedentary than rural women [12].

We hypothesise that urban, black African females have a high prevalence of obesity and metabolic disease both of which are associated with physical inactivity. Therefore, the aim of this study was three-fold: (i) to describe patterns of physical activity in a middle-aged cohort of urban black South African women who have recently been shown to have a high prevalence of metabolic syndrome and related disorders [16]; (ii) to examine the association between socio-economic status and physical activity patterns in this cohort; and (iii) to determine if physical activity is associated with anthropometry and metabolic variables.

## Methods

### Study population

The study design was cross sectional, and included black African women, who have previously been shown to have a high prevalence of metabolic syndrome, living in an urban setting (Soweto, Johannesburg) [16]. The participants were caregivers from the Birth to Twenty cohort study (Bt20), which began in 1990 when 3273 participants were enrolled to investigate the health and development of children [17]. Bt20 also monitored the health of their biological mothers or caregivers. Participants for this study were included if they answered the Global Physical Activity Questionnaire (GPAQ) and excluded if they were from other ethnic groups, or younger than 18 years of age. The final sample size was 977 which represented 78% of the black African caregivers from the original population of caregivers, and of whom 71.8% were biological mothers. Ethical clearance was obtained from the Human Research Ethics Committee at the University of the Witwatersrand (M010556).

### Anthropometric measures

Total body weight (kg) was measured to the nearest 0.1 kg using a digital weighing scale (Dismedinc., Anjou, Canada) and standing height was measured to the nearest mm using a wall stadiometer (Holtain Ltd., Crosswell, UK). The participants wore minimal clothing and did not have shoes on during the measurements. Trained research assistants conducted the measurements, and the coefficients of variation for body weight and standing height measurements were both <1%.

Body mass index (BMI, kg.m^−2^) was calculated and classified as normal (≥18.5 and < 25 kg.m^−2^), overweight (≥25 and < 30 kg.m^−2^) or obese (≥30 kg.m^−2^) [18]. Using a flexible, but inelastic measuring tape, a measurement of the waist circumference was taken at the narrowest part of the trunk, horizontally, while the participants were standing with arms at the side, relaxed abdomen, and feet together [18]. Similarly, the hip circumference measurement was taken at the widest circumference of the proximal thigh, just under the fold of the gluteus, with feet separated slightly [18]. Whole body fat and fat free soft tissue mass were measured using dual-energy X-ray absorptiometry (DXA) (Hologic QDR 4500A, software version 11.2, Hologic Inc., (Bedford, Massachusetts, USA)).

### Blood pressure

The Omron M6 (version HEM-7001-E, Omron, Kyoto, Japan) was used to record brachial blood pressure (BP). Three measurements were taken with the participant in the seated position with the cuff around the right upper arm, supported at the level of the heart [18]. The average of the last two BP measurements was recorded.

### Biochemical analyses

Fasting blood samples were collected and centrifuged to obtain plasma and serum samples. Aliquot samples were stored at −70°C until assayed for lipid profile and glucose concentrations using an automated methodology (Randox Laboratories Ltd., County Antrim, UK). Insulin was measured (IMMULITE® 1000 Chemiluminescent Technology) and insulin resistance was quantified using the Homeostasis Model Assessment (HOMA) technique [19].

### Metabolic disease risk factors

Metabolic disease risk factors were defined as systolic BP ≥130 mm Hg, diastolic BP ≥ 85 mm Hg, fasting blood glucose ≥5.6 mmol.L^−1^, triglycerides (TG) ≥ 1.7 mmol.L^−1^,high density lipoprotein cholesterol (HDL-C) <1.3 mmol.L^−1^, low density lipoprotein cholesterol (LDL-C) ≥ 3 mmol.L^−1^, and total cholesterol (TC) ≥ 5 mmol.L^−1^ [20]. Central obesity was defined as a waist circumference ≥ 80 cm [20]. Metabolic syndrome was defined using the harmonised guidelines [20].

### Physical activity questionnaire

The Global Physical Activity Questionnaire (GPAQ), developed for global physical activity surveillance, was completed via interview to obtain self-reported physical activity [21]. The hours of physical activity and metabolic-equivalent (MET) values per activity were multiplied together to give MET minutes per week. Moderate activity was allocated a MET value of four and vigorous physical activity a value of eight as outlined in the World Health Organisation (WHO) guidelines [22]. Total moderate-vigorous physical activity (MVPA) in minutes per week (mins/wk) were calculated from the accumulative occupation, travel-related and leisure time physical activity. Walking for travel was analysed individually as it was the most common form of physical activity noted in the study population. Minutes per week of work and leisure time physical activity were combined. Sitting time (mins/wk) was used as a proxy for sedentary behaviour.

The participants were also grouped according to the GPAQ criteria, into GPAQ active or GPAQ inactive categories [22]. GPAQ active was defined as taking part in: moderate physical activity for a total of 150 minutes per week (≥5 days per week); or vigorous physical activity for 60 minutes per week (≥3 days per week); or 600 metabolic minutes per week [≥5 days moderate-vigorous physical activity (MVPA)] [22]. Participants who did not meet these criteria were classified as inactive.

### Socio-economic status

A questionnaire was used to assess household socio-economic status (SES) [23]. The questionnaire was based on the ownership of twelve household commodities: electricity, television, radio, motor vehicle, refrigerator, washing machine, telephone, video machine, microwave, analog television channel decoder (MNET), satellite television (DSTV), and mobile phone. The twelve household commodities were ranked in order of value and an overall SES score was then calculated using the ranks. The overall SES score ranged from 0 to 78.Television and motor vehicle ownership were stratified and analysed separately from the other household possessions as they are recognised as stimulators of sedentary behaviour [24, 25].

### Statistical methods

Statistica version 12 (StatSoft, Tulsa, OK, USA) was used to carry out the statistical analyses [26]. Prevalence levels for observed metabolic risk were determined using the harmonised guidelines [20]. Multiple imputation was used in dealing with missing body composition (fat mass, and fat free soft tissue mass) and metabolic outcome variables (fasting blood glucose, fasting insulin, HOMA, HDL, LDL, triglycerides, and total cholesterol). Multiple imputation with chained equations was then performed, using linear regression as the imputation model.

Dependent variables that were not normally distributed (systolic BP, fasting blood glucose, fasting insulin, HOMA, LDL, HDL, triglycerides) were log transformed to normality. Model assumptions of normality and constant variance were tested using Q-Q plots, and plots of residuals versus predicted values, respectively. Data are presented as mean ± SD if normally distributed; otherwise median [interquartile range (IQR)] is presented.

T-tests were used to compare body composition and metabolic outcomes between GPAQ active and inactive subjects, individuals who presented with the extremes of MVPA (above 90th and below 10th percentile) and physical activity between subjects who owned or did not own a motor vehicle or TV. A cluster variable was created using sitting time with total MVPA and then using ANOVA to compare metabolic and body composition measures between subjects in: highest tertiles for MVPA and sitting time (n = 129), lowest tertile for MVPA and sitting time (n = 111), highest tertile for MVPA and lowest tertile for sitting time (n = 77) and lowest tertile for MVPA and highest tertile for sitting time (n = 90). Differences between tertiles of walking for travel MVPA were explored using an ANOVA for body composition measures and metabolic outcomes, and Tukeys test, which takes into account multiple comparisons was used for the post-hoc analysis.

Multiple linear regression analyses were performed to determine if any of the physical activity variables were associated with the body composition and metabolic variables. The dependent variables were: total body fat mass, fat-free soft tissue mass, waist circumference, HOMA, fasting blood glucose, fasting insulin, total cholesterol, LDL, HDL and triglyceride, and systolic and diastolic blood pressures. The independent variables to include in the initial models were chosen based on scientific plausibility and for the models with anthropometric measures as the dependent variable these were: age, SES, fat mass (for the waist model only), sitting time, total MVPA, work MVPA, leisure MVPA and walking for travel. The multiple linear regression models that used metabolic measures as the dependent variables included the following independent variables: age, SES, total body fat mass, fat-free soft tissue mass, waist circumference, sitting time, total MVPA, work MVPA, leisure MVPA and walking for travel MVPA. Before performing multiple linear regression, simple univariate regressions were used to determine which of the independent variables listed above were associated with the various dependent variables and these were included in the regression models. The final models were also checked for multicollinearity using the Variance Inflation factor (VIF), but no multicollinearity was noted (all VIFs < 3.0). The results of the regression models are reported as standardised β values to facilitate direct comparisons of the strengths of the associations. Significance was accepted at a level of p < 0.05.

## Results

### Subject characteristics, anthropometric measures and metabolic outcomes

The mean age of this cohort was 41.0 ± 7.84 years, with a high mean BMI (30.3 ± 6.73 kg.m^−1^) and waist circumference (86.9 ± 13.2 cm) (Table 1). The percentage fat mass was also high (40.8 ± 7.38%). Fasting blood glucose levels were slightly raised, whilst insulin and HOMA levels were normal, and HDL levels were low (0.99 (IQR 0.78-2.00)), as were total cholesterol, LDL and triglyceride levels. Blood pressure values were within normal limits.

**Table 1:** **Subject characteristics, body composition and metabolic outcomes of middle aged black African women from the birth to twenty caregiver cohort**

Characteristics	n	Mean ± SD or median (IQR)
**Age (years)**	964	41.0 ± 7.84
**Body mass index (kg.m** ^**−2**^ **)**	977	30.3 ± 6.73
**Waist circumference (cm)**	964	86.9 ± 13.2
**Hip circumference (cm)**	964	112 ± 13.6
**Waist-to-hip ratio**	964	0.78 ± 0.08
**Fat mass (kg)**	655	28.9 ± 10.7
**Fat free soft tissue mass (kg)**	655	38.1 ± 5.96
**Fat percentage (%)**	655	40.8 ± 7.38
**Fasting blood glucose (mmol.L** ^**−1**^ **)**	551	5.09 (4.59-5.80)
**Fasting insulin (pmol.L** ^**−1**^ **)**	352	6.74 (4.46-9.14)
**Homeostasis model insulin resistance**	330	1.48 (0.96-2.11)
**High density lipoprotein cholesterol (mmol.L** ^**−1**^ **)**	462	0.99 (0.78-2.04)
**Low density lipoprotein cholesterol (mmol.L** ^**−1**^ **)**	462	1.31 (0.88-1.77)
**Triglycerides (mmol.L** ^**−1**^ **)**	463	0.95 (0.71-1.67)
**Total cholesterol (mmol.L** ^**−1**^ **)**	463	3.44 ± 1.77
**Systolic blood pressure (mm Hg)**	925	112 (103–126)
**Diastolic blood pressure (mm Hg)**	925	76.4 ± 12.5

Data presented as mean ± SD or median (interquartile range (IQR)).

### Physical activity

**Figure 1:**
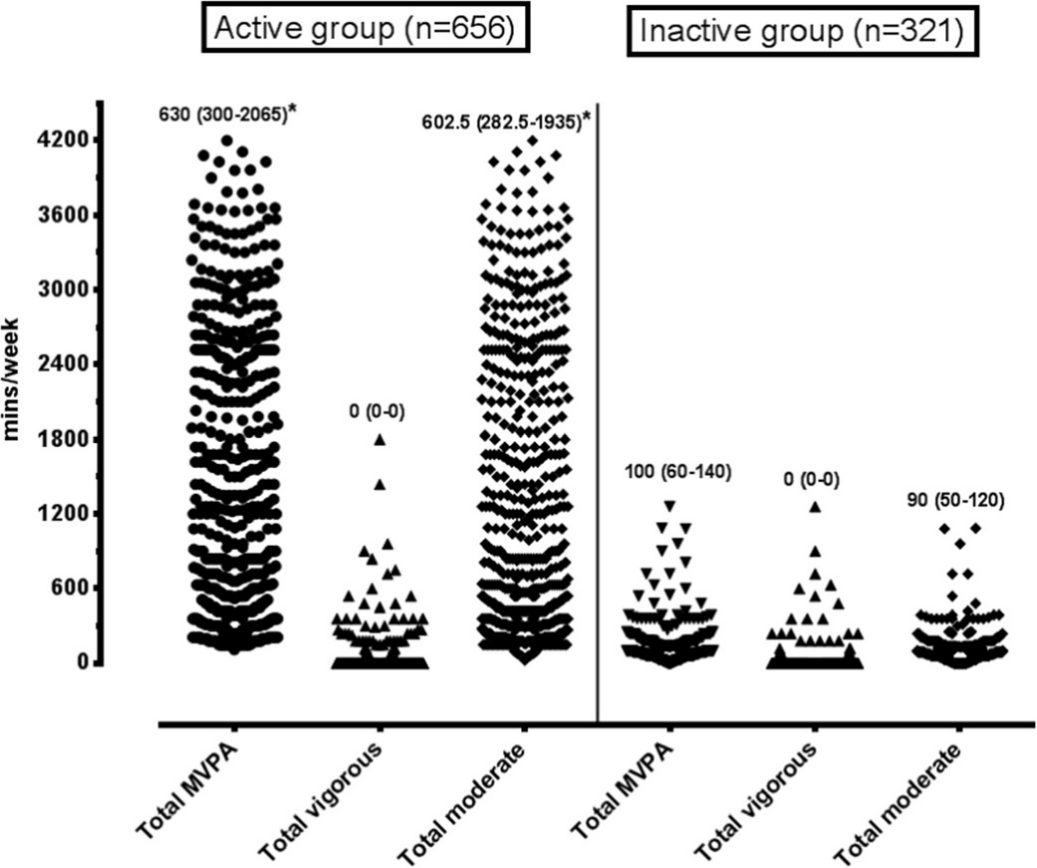
**Comparative diagram of cumulative weekly physical activity for total MVPA, total vigorous and moderate physical activity between GPAQ active and GPAQ inactive groups.** *p<0.05 when compared to matching physical activity domain.

**Figure 2:**
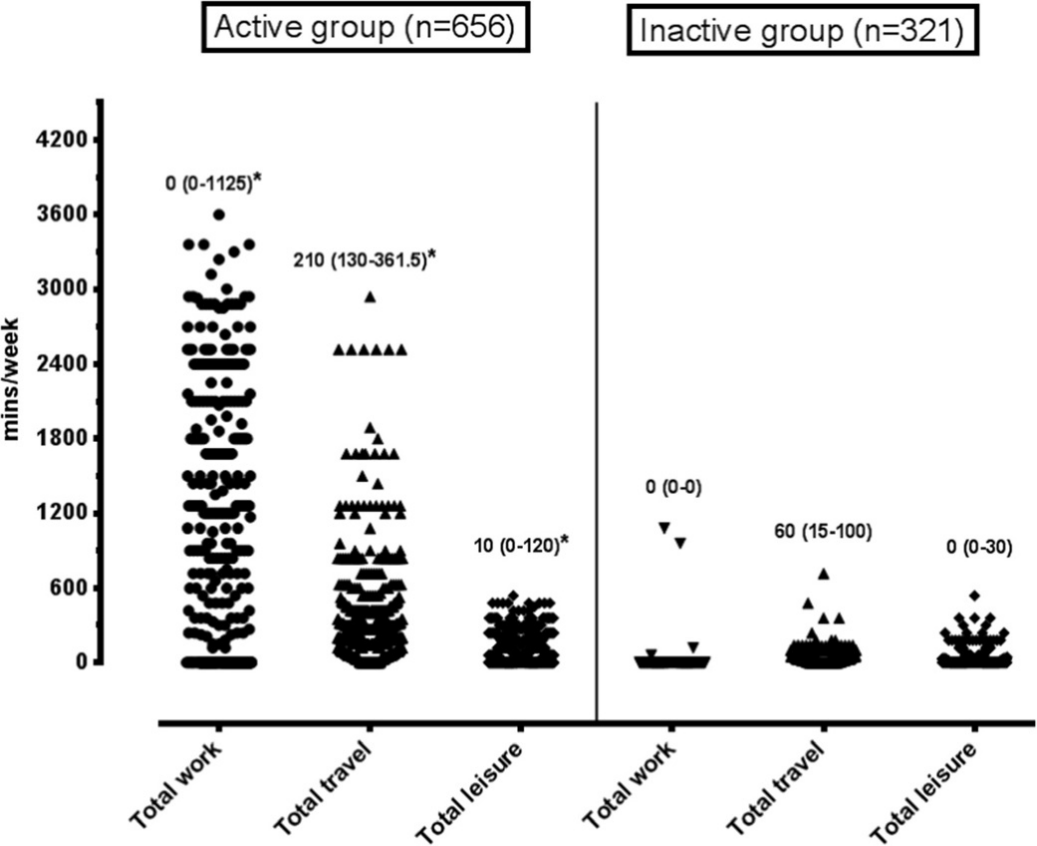
**Comparative diagram of cumulative weekly physical activity in the occupation-, transport- and recreational-time between GPAQ active and GPAQ inactive groups.** *p<0.05 when compared to matching physical activity domain.

Physical activity data was obtained for 977 participants, 67% of whom were classified as physically active according to the Global Physical Activity Questionnaire (GPAQ) criteria. When using the WHO criteria, 75% of the participants met the minimum recommended guidelines for attainment of 150 minutes of moderate activity or 75 minutes of vigorous weekly activity [22]. Age was not significantly different between the physical activity groups. All domains of physical activity, except vigorous PA (p = 0.64), were significantly higher (all p < 0.001) in the GPAQ active group compared to the GPAQ inactive group (Figures 1 and 2). The median sitting time for the whole group was 3 hours a day, and was not significantly different between the activity groups (GPAQ active: 3 (IQR: 2–5) vs. GPAQ inactive: 3 (IQR: 1.5-4) hours per day).

### Association between physical activity and socio-economic status (SES)

Household SES score was inversely associated with time spent walking for travel (r = −0.10; p < 0.01), but was not associated with any other activity variable. Walking for travel was divided into tertiles for analysis, lowest tertile: n = 322, 0–90 minutes per week; middle tertile: n = 316, 90–210 minutes per week; and highest tertile: n = 339, ≥210 minutes per week. Participants in the lowest tertile for walking for travel had a significantly higher household SES score (6.48 ± 2.29) compared to women in the middle (6.08 ± 2.28, p < 0.05) and highest (5.81 ± 2.37, p < 0.001) tertiles.

### Sedentary promoting assets and physical activity

Time spent walking for travel was significantly higher in the women who did not own a motor vehicle compared to those who did (p < 0.01), and significantly higher in the women who did not own a television compared to those who did (p = 0.001) (Table 2). Leisure time physical activity was significantly higher in the women who owned a motor vehicle compared to those who did not (p < 0.01). Women who owned a television reported less MVPA minutes/wk (p < 0.01) and total moderate PA (p < 0.01), than women who did not own a television (Table 2). Sitting time in the GPAQ active group, 1260 (IQR: 840–2100) minutes per week, did not differ significantly from the inactive group, 1260 (IQR: 630–1680) minutes per week.

**Table 2:** **Physical activity domains of participants who do and do not own a motor vehicle or television (TV) ownership**

Physical activity domains ^*^	Motor vehicle and TV ownership					
	No motor vehicle (n = 601)	Motor vehicle (n = 211)	p-value	No TV (n = 738)	TV (n = 74)	p-value
**Total moderate-vigorous physical activity**	400 (150–1320)	315 (150–1260)	0.53	615 (240–2160)	360 (150–1260)	0.004
**Total moderate physical activity**	360 (140–1260)	300 (120–1200)	0.46	615 (210–2100)	315 (120–1230)	0.002
**Total vigorous physical activity**	0 (0–0)	0 (0–0)	0.16	0 (0–0)	0 (0–0)	0.41
**Total work (moderate-vigorous physical activity)**	0 (0–330)	0 (0–0)	0.39	0 (0–720)	0 (0–0)	0.06
**Total walking for travel**	150 (60–300)	120 (40–240)	0.003	210 (120–420)	140 (60–280)	0.001
**Total leisure (moderate-vigorous physical activity)**	0 (0–120)	30 (0–120)	0.004	0 (0–180)	0 (0–120)	0.39
**Work-leisure (moderate-vigorous physical activity)**	60 (0–960)	90 (0–760)	0.38	120 (0–1680)	60 (0–60)	0.16
**Sitting time**	1260 (840–2100)	1260 (840–1680)	0.26	1260 (840–1680)	1260 (840–2100)	0.94

^*^Units are minutes/week expressed as median (interquartile range).

### Association between physical activity, and anthropometric measures and metabolic outcomes

The prevalence of overweight was 29.2% (95% CIs 26.3, 32.0), obesity 48.0% (95% CIs 44.9, 51.1), 66.2% of the women had a waist circumference ≥80 cm (95% CIs 63.2, 69.1), diabetes was observed in 29.0% (95% CIs 25.2, 32.8) of the cohort and the prevalence of metabolic syndrome in this cohort was 40.0% (95% CIs 35.5, 44.6). Table 3 shows that the absolute levels of the anthropometric and metabolic variables did not differ between the GPAQ active and inactive groups. In addition, there were no significant differences between any of the anthropometric or metabolic variables when comparing women in the 90th (n = 99) and 10th (n = 101) percentiles for MVPA (data not shown). Sitting time was not different between those with metabolic syndrome and those without (data not shown). The exploratory cluster variable also failed to show any significant differences between cluster groups for BMI, waist circumference, body fat, systolic blood pressure, diastolic blood pressure, fasting blood glucose, HDL, LDL, total cholesterol, and triglycerides (data not shown).

**Table 3:** **Comparison of metabolic risk outcomes and body composition between active and inactive black South African women based on multiple imputation**

		GPAQ inactive		GPAQ active
**Dependent variable**	**N**	**Mean (95** **%** **CI)**	**N**	**Mean (95** **%** **CI)**
**Fasting blood glucose (mmol.L** ^**−1**^ **)**	309	5.13 (4.92, 5.33)	630	5.32 (5.10, 5.53)
**Fasting insulin (pmol.L** ^**−1**^ **)**	306	7.14 (6.31, 8.09)	625	6.78 (5.96, 7.71)
**High density lipoprotein cholesterol (mmol.L** ^**−1**^ **)**	306	1.27 (1.15, 1.40)	618	1.38 (1.23, 1.55)
**Low density lipoprotein cholesterol (mmol.L** ^**−1**^ **)**	306	1.26 (1.18, 1.35)	618	1.18 (1.12, 1.24)
**Total cholesterol (mmol.L** ^**−1**^ **)**	306	3.52 (3.3, 3.75)	618	3.30 (3.10, 3.50)
**Triglycerides (mmol.L** ^**−1**^ **)**	306	1.06 (0.98, 1.14)	618	1.10 (1.03,1.17)
**Systolic blood pressure (mm Hg)**	305	115 (112, 117)	620	114 (112, 117)
**Diastolic blood pressure (mm Hg)**	305	76 (74, 77)	620	77 (76, 78)
**Fat mass (kg)**	309	29.42 (28.21, 30.62)	635	29.01 (28.15, 29.87)
**Fat free soft tissue mass (kg)**	309	38.15 (37.47, 38.84)	635	38.34 (37.82, 38.85)
**Waist circumference (cm)**	316	86.06 (84.63, 87.49)	650	87.25(86.22, 88.28)
**Body mass index (kg.m** ^**−2**^ **)**	321	30.22 (29.48, 30.97)	656	30.30 (29.78, 30.81)

Data expressed as mean (95% CIs).

GPAQ: Global physical activity questionnaire.

The results of the multiple regression analyses using observed data can be viewed in Additional file 1. Using imputed data, multiple linear regression analysis demonstrated that sitting time was positively associated with HDL, triglycerides, and diastolic blood pressure (Table 4). The relationship sitting time and HDL was confounded by the interaction between triglycerides and HDL. Thus, when triglycerides were added to the HDL model, sitting time showed an inverse, non-significant relationship with HDL (beta coefficient (β): −2.13, p = 0.67). However, when triglycerides were removed from the HDL model, sitting time showed a positive association with HDL (β: 0.000002, p = 0.02). Inverse associations were observed between total MVPA and insulin, in addition to walking for travel and total cholesterol, whilst work MPVA was positively associated with fat free soft tissue mass. Age was found to be positively associated with body fat, waist circumference, fasting blood glucose, total cholesterol, LDL, triglycerides, systolic and diastolic blood pressure, whilst SES was positively associated with body fat mass and fat free soft tissue mass. Waist circumference was associated with fasting glucose, fasting insulin, LDL, total cholesterol, systolic and diastolic blood pressure (Table 4). No independent variables were found to correlate with HOMA levels following the regression analysis.

**Table 4:** **Multiple linear regression models for anthropometric and metabolic variables using imputed data**

Dependent variable	N	Independent variables	Coefficients ( 95% CI)‡	Beta coefficient† (p-value)	Adjusted R ^2^(p-value)
**Fasting glucose**	918	Age	0.001 (0.0004, 0.002)	0.10 (0.003)	0.02 (<0.001)
		Waist	0.0007 (0.0003, 0.001)	0.11 (0.001)	
**Fasting insulin**	916	Age	−0.002 (−0.005, 0.002)	−0.07 (0.04)	0.13 (<0.001)
		Waist	0.006 (0.004, 0.007)	0.35 (<0.001)	
		Total MVPA	−0.00002 (−0.00004, −0.000004)	−0.11(<0.001)	
**High density lipoprotein cholesterol**	914	Age	−0.001 (−0.003, 0.001)	−0.05 (0.16)	0.04 (<0.001)
		Waist	−0.003 (−0.004, −0.002)	−0.16 (<0.001)	
			0.00002 (0.000003, 0.00003)	0.08 (0.02)	
**Low density lipoprotein cholesterol**	917	Age	0.006 (0.002, 0.009)	0.28 (<0.001)	0.13 (<0.001)
		Waist	0.002 (0.0005, 0.004)	0.18 (<0.001)	
**Total cholesterol**	917	Age	0.04 (0.02, 0.07)	0.25 (<0.001)	0.09 (<0.001)
		Waist	0.009 (−0.009, 0.03)	0.09 (0.007)	
		Walking for travel	−0.0003 (−0.0009, 0.0002)	−0.08 (0.01)	
**Triglycerides**	921	Age	0.002 (−0.0002, 0.004)	0.08 (0.01)	0.02 (<0.001)
		Sitting time	0.00002 (0.000001, 0.00004)	0.12 (<0.001)	
**Systolic blood pressure**	925	Age	0.002 (0.002, 0.003)	0.24 (<0.001)	0.10 (<0.001)
		Waist	0.0009 (0.0006, 0.001)	0.17 (<0.001)	
**Diastolic blood pressure**	912	Age	0.21 (0.10, 0.30)	0.13 (<0.001)	0.08 (<0.001)
		Waist	0.20 (0.14, 0.26)	0.21 (<0.001)	
		Sitting time	0.001 (0.0002, 0.002)	0.08 (0.01)	
**Body fat**	925	Age	201.03 (112, 291)	0.15 (<0.001)	0.03 (<0.001)
		SES score	46.4 (9.82, 82.9)	0.08 (0.009)	
**Fat free soft tissue mass**	925	Age	1.11 (−46.4, 48.6)	0.002 (0.96)	0.01 (0.098)
		SES score	15.31 (−4.00, 34.62)	0.05 (0.12)	
		Work MVPA	0.46 (−0.001, 0.92)	0.06 (0.05)	
**Waist circumference**	925	Age	0.35 (0.25, 0.45)	0.21 (<0.001)	0.04 (<0.001)

‡Unstandardised model coefficients.

†Standardised model coeffcients.

MVPA: moderate-vigorous physical activity.

## Discussion

The aim of this cross-sectional study was to determine the physical activity patterns of a cohort of middle-aged black women from Soweto, Johannesburg, who have previously been shown to be at high risk for metabolic disease. In this cohort of women in whom 67.0% were classified as physically active, the prevalence of obesity was 48.0%. This study is one of only a few to measure sedentary time in black South African women, and shows that despite a high level of physical activity and relatively low sitting time, metabolic disease risk is still high. Walking for travel significantly contributed to weekly physical activity, and was inversely associated with sedentary promoting assets including motor vehicle and television ownership.

The WHO defines being sufficiently active as accumulating a minimum of 150 minutes of moderate activity or 75 minutes of vigorous activity per week, however, this method does not take into account the various domains of physical activity [22]. Physical activity in developed countries typically encompasses a greater contribution from leisure time activity, whereas in developing countries work- and travel-related physical activity are the major contributors to daily energy expenditure [27]. In addition, physical inactivity is higher in more affluent countries than lower income countries [28]. However, longitudinal data from Brazil shows that there has been an increase in physical activity in the lower socioeconomic stratum over a 5 year period, suggesting a shift in physical activity patterns in low and middle income countries [29]. Using the GPAQ criteria which also takes into account the number of days per week of PA, the majority (67.0%) of women in this study were classified as physically active. This is comparable to the global level of physical activity in women (66.1%), in a study which also highlighted the lack of physical activity data from low and middle income African and Asian countries [28]. However, the physical activity range in our study falls into the higher end of the range reported in other studies of black South African women (45.2-70.8%) [30, 31]. In the study by Alberts et al. 45.2% (age-standardized) of rural women were classified as physically active at home using a lifestyle questionnaire [30], while the THUSA study found that 70.8% of rural women were physically active using a physical activity index which stratified the groups of physical activity in tertiles [31]. In comparison to these two South African studies, most of the women in the current study performed walking for travel (89.0%) whereas the percentage of subjects commuting by walking was lower in the THUSA study (27.4%) and the study by Alberts et al. (16%). However, it should be noted that the methods used in these studies for assessing physical activity were different to those used in the current study, making comparisons difficult.

This is one of the first studies to quantify daily sitting time (excluding sleep time), a proxy for sedentary time [32], in a large female African population. Evidence from studies suggest that sitting time is associated with both obesity and other metabolic diseases [33, 34]. In our study sitting time was positively associated with triglycerides and diastolic blood pressure, which has also been observed in other studies [33, 35]. In the current study 50.0% of the women reported sitting for 3 or more hours a day which is comparable to studies from India, China, and Brazil who report 3.5, 4, and 4.5 hours of sitting per day, respectively [36, 37]. In our study, sitting time was not different between the active and inactive groups, suggesting that in this population, the amount of time spent sitting is independent of physical activity, and should be investigated as a distinct entity. This opinion is also shared by Bankoski et al. [38] and Chau et al. [39] who found that time spent sedentary was strongly related to the risk of metabolic disease independent of the time spent being physically active. Recent findings also show that patterns of sedentary behaviour varies by life domains such as television watching, personal computer use, and travel time [35].

Previous studies have identified motor vehicle ownership as a sedentary promoting asset [24, 38]. Held et al. found that participants who owned a motor vehicle had an increased risk of myocardial infarction [38]. Similarly the review by Douglas et al. emphasises the integral role of car ownership in the increasing prevalence of physical inactivity and obesity in countries with low levels of active transport [24]. The current growth of the South African economy has resulted in motor vehicles becoming more affordable to a larger proportion of the population, as a result of which, vehicle ownership is increasing in urban settings such as Soweto [39]. In our study, sitting time was not different between the women who owned motor vehicles and those that did not; however the women who did not own a motor vehicle walked significantly more and performed significantly less leisure time physical activity than the women who did own a motor vehicle. This data suggests that the negative effect of car ownership on walking may be counteracted by an increased amount of leisure time physical activity. Leisure time physical activity has previously been shown to be associated with SES [40], so the role of SES and time spent in different sedentary life domains in determining physical activity patterns must also be considered [41].

The domains of physical activity in developing countries favour travel- and occupation-related physical activity, which was confirmed in our findings [27]. Importantly, our study has provided data on the different domains of physical activity, which has only been investigated in a small number of African countries [42]. Our findings show that the majority of the women (89.5%) did not perform vigorous activity and that walking and occupational physical activities were the domains with the highest contribution (34% from walking and 56% from occupation related physical activity) to overall physical activity. A similar pattern of physical activity is evident in African countries such as Eritrea, Cameroon, Mali, and Mauritania, where active commuting contributes more than 50% to total daily physical activity [42]. Our study showed a positive association between activity in the work domain and fat free soft tissue mass, suggesting that physical activity may have a role in improving the overall health profile of this ageing population of women, despite them having a high body fat percentage. A recent study concurred with this finding, showing that exercise improved physical fitness and lean mass, but did not result in significant body fat or lipid profile changes following a 24 week intervention of strength and endurance training [43]. Studies have also shown that participation in regular physical activity is the best means of preventing the effects of sarcopenia on the physical functioning of ageing individuals [44, 45]. Leisure time activity contributes the most to overall physical activity in developed countries [5]. A recent systematic review of the health benefits of walking as a means of transport confirms that there may be positive effects on hypertension and type 2 diabetes mellitus with longer duration walking [46].

Our findings showed that total MVPA was inversely associated with fasting insulin, indicating that being physically active has a role in enhancing insulin sensitivity. These results correspond with South African studies which showed that physically active urbanised black women had lower serum insulin levels than inactive women [13], subsequently resulting in higher levels of insulin sensitivity [47]. These studies imply that meeting the recommended guidelines for physical activity may have long-lasting effects on insulin sensitivity.

This study used the GPAQ to measure physical activity. The GPAQ is cost-effective and has been validated for use in developing countries [27, 48]. The reliability of the GPAQ has been tested in South Africa, with the results showed acceptable Kappa statistics, ranging from 0.66 (93.9% agreement) to 0.78 (89.3% agreement) across the domains of physical activity [21]. However, the major challenge of using this instrument is that it assesses self-reported physical activity which may lead to an overestimation of weekly activity [49]. Secondly, the GPAQ is not as sensitive as objective measurements of physical activity; however the GPAQ is still a useful tool for physical activity surveillance. Our South African data compares well with data from other African countries [42], such as Mozambique, Niger and Malawi who attain the majority of their activity from the work domain, ranging from 60-75% of total MVPA [42].

Another key drawback of this study is that it was cross sectional and therefore causality cannot be determined. We did not find an association between any of the physical activity domains and anthropometric measures or metabolic outcomes using a variety of statistical analyses. In an urban dwelling Cameroon population of women, lower amounts of MVPA was performed compared to ours (94 vs. 119 MVPA mins/per day) and a negative association was observed between physical activity and prevalence of metabolic syndrome [50]. Cook et al. found that more than half of the women (55.2%) in rural South Africa performed more than the recommended 10000 steps per day, and that ambulation reduced obesity risk in rural South Africans [15]. A reasonable assumption for the difference could be in the use of self-reported questionnaires in our study compared to objective measurement of physical activity used in the other studies. However another reason for the difference could be that the prevalence of obesity was also lower in the Cook study (27.1%) and similarly, the mean BMI of the women in the Cameroon study ranged from 23.7 in the highest quartile of physical activity to 28.3 in the lowest.

## Conclusions

We have shown that despite the majority of urban dwelling black South African women being classified as physically active, there is still a high prevalence of obesity and metabolic disease in this population. As in other developing countries, the majority of time spent in physical activity is in the form of walking for travel, however the intensity of this activity is not known. Thus, we recommend that future research aims to determine whether the intensity of this walking modifies the level of cardiometabolic risk factors in this population. This study showed that sitting time was positively associated with serum triglyceride levels and diastolic blood pressure, whilst total MVPA was inversely associated with serum insulin levels. This data implies that future intervention studies for cardiometabolic disease prevention in urban African populations must aim to reduce daily sitting time and increase total MVPA.

## Electronic supplementary material

Additional file 1: **Multiple regression models for anthropometric and metabolic variables using observed data.** (PDF 92 KB)
