# Supplementary material for: Patterns, levels and correlates of self-reported physical activity in urban black Soweto women
Source: BMC Public Health. 2014 Sep 8;14:934. doi: 10.1186/1471-2458-14-934 (PMC4176558; doi:10.1186/1471-2458-14-934)
Supplement: Supplementary file 1 — Additional file 1: Multiple regression models for anthropometric and metabolic variables using observed data. (PDF 92 KB) [file 12889_2013_7063_MOESM1_ESM.pdf]

Additional file 1: Multiple regression models for anthropometric and metabolic variables using observed data

Dependent variable	N	Independent variables	Coefficients (95% Confidence Interval)‡	Beta Coefficients† (p-value)	Adjusted R^2 (p-value)
**Fasting blood glucose**	530	Age	0.0013 (0.0002, 0.0025)	0.09 (0.03)	0.02 (0.002)
		Waist circumference	0.0008 (0.00001, 0.0015)	0.10 (0.03)	
**Fasting insulin**	310	Age	-0.003 (-0.006, 0.0009)	-0.08 (0.15)	0.09
		Waist circumference	0.007 (0.004, 0.009)	0.31 (<0.001)	(<0.001)
**High density lipoprotein cholesterol**	455	Age	-0.002 (-0.006, 0.0011)	-0.06 (0.18)	0.02 (0.006)
		Waist circumference	-0.003(-0.005, -0.0004)	-0.12 (0.02)	

**Low density lipoprotein cholesterol**	455	Age	0.007 (0.004, 0.009)	0.26 (<0.001)	0.11
		Waist circumference	0.002 (0.0008, 0.004)	0.14 (0.002)	(<0.001)
**Total cholesterol**	456	Age	0.05 (0.03, 0.07)	0.23 (<0.001)	0.07
		Waist circumference	0.008 (-0.005, 0.02)	0.06 (0.23)	(<0.001)
		Walking for travel	-0.0004(-0.0009, 0.00008)	-0.09 (0.05)	
**Systolic blood pressure**	602	Age	0.002 (0.0015, 0.003)	0.23 (<0.001)	0.10
		Waist circumference	0.0009 (0.0006, 0.001)	0.17 (<0.001)	(<0.001)
**Diastolic blood pressure**	599	Age	0.21 (0.11, 0.32)	0.13 (<0.001)	0.07
		Waist circumference	0.20 (0.13, 0.26)	0.20 (<0.001)	(<0.001)
		Sitting time	0.001 (0.0002, 0.002)	0.08 (0.01)	
**Fat mass**	636	Age	238 (139, 337)	0.18 (0.001)	0.05
		SES score	66.8 (25.5, 108)	0.12 (0.002)	(<0.001)

**Fat free soft tissue mass**	636	Age	13.2 (-43.4, 69.8)	0.02 (0.65)	0.02 (<0.001)
		SES score	27.6 (3.93, 51.2)	0.09 (0.02)	
		Work MVPA	0.69 (0.12, 1.26)	0.09 (0.02)	
**Waist circumference**	925	Age	0.35 (0.25, 0.45)	0.21 (<0.001)	0.04 (<0.001)
